# Comparative shotgun proteomics analysis of wheat gluten proteins digested by various peptidases

**DOI:** 10.1016/j.crfs.2025.101095

**Published:** 2025-05-26

**Authors:** Christine Kaemper, Johanna Mossburger, Manuel Geyer, Lorenz Hartl, Sabrina Geisslitz, Katharina Anne Scherf

**Affiliations:** a Department of Bioactive and Functional Food Chemistry, Institute of Applied Biosciences, Karlsruhe Institute of Technology (KIT), 76131, Karlsruhe, Germany; b Technical University of Munich, TUM School of Life Sciences, 85354, Freising, Germany; c Leibniz Institute for Food Systems Biology at the Technical University of Munich, 85354, Freising, Germany; d Bavarian State Research Center for Agriculture, Institute for Crop Science and Plant Breeding, 85354, Freising, Germany; e Technical University of Munich, TUM School of Life Sciences, Professorship of Food Biopolymer Systems, 85354, Freising, Germany

**Keywords:** Bottom-up proteomics, Enzymatic hydrolysis, Gliadin, Glutenin, Label-free quantification, *Triticum aestivum*, Untargeted mass spectrometry

## Abstract

The wheat flour proteome is a complex mixture of non-gluten and gluten proteins. The large number of repetitive sequences, special amino acid composition and similarity of gluten protein isoforms pose a major challenge in bottom-up proteomics. The standard peptidase used in shotgun proteomics is trypsin, which may not be suitable for all wheat proteins. In this study, flour extracts of nine common wheat genotypes were digested with the peptidases trypsin, chymotrypsin, thermolysin, pepsin and the combination of trypsin and chymotrypsin. The results showed large differences for the number of identified peptides. With 4115 peptides, trypsin resulted in by far the most peptide identifications, followed by thermolysin with 1421 peptides. However, this no longer applied at protein level. Most metabolic protein groups (673) were identified with trypsin. Among the gluten protein groups, however, around 130 were identified with each peptidase. The ω-gliadins were detected with all peptidases except trypsin. A comparison with quantitative RP-UHPLC-UV results showed that there was the greatest overlap after thermolysin digestion. Otherwise, there was no great similarity between the different peptidases, which is why their results cannot be compared with one another. The sequence coverage of gluten proteins was 51 % after thermolysin digestion, 27 % after trypsin digestion and 61 % when all peptidases were evaluated together. The results showed that digestion with various peptidases provides a more detailed picture of the wheat proteome. Some wheat protein groups could only be identified with certain peptidases, which is important if these protein groups are to be studied in more detail.

## Highlights

• Identification and quantitation of gluten protein types depend on the peptidase.
• Most peptides and protein groups were identified after trypsin digestion.
• Gluten protein type of ω-gliadins was not detected after trypsin digestion.
• Quantitative data of different peptidases cannot be compared.
• Multiple digestion increases protein sequence coverage.

## Graphical abstract

**Figure:**
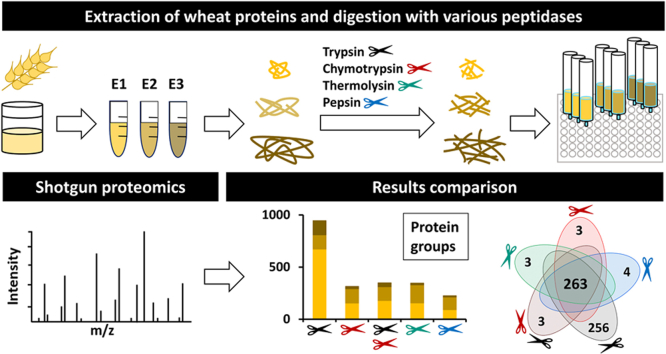

## 1 Introduction

Common wheat (*Triticum aestivum* L.) is one of the most important food crops in the world. Its baking properties are unique and enable the production of leavened bread and a wide variety of other baked goods. These special baking properties can be attributed to the proteins present in wheat flour. The protein content in wheat flour varies in a wide range from about 8 to 16 % depending on the cultivar and the growing conditions (Geisslitz et al., 2018; Hajas et al., 2018). The proteins are divided into about 20 % of metabolic proteins and 80 % of storage proteins, the so-called gluten proteins. These are mobilized during germination to help the seedling grow. Gluten proteins are subdivided into gliadins and glutenins. Gliadins are further categorized into α-, γ-, ω1,2- and ω5-gliadins based on their different mobility in acidic polyacrylamide gel electrophoresis (PAGE). Separated by sodium dodecyl sulphate (SDS)-PAGE, glutenins are differentiated according to their molecular weight into low-molecular-weight glutenin subunits (LMW-GS) and high-molecular-weight glutenin subunits (HMW-GS) (Wieser et al., 2022). Gliadins are responsible for the viscosity/extensibility, while glutenins contribute to the strength/elasticity of the dough (Wieser et al., 2022).

Common wheat is a hexaploid plant, with seven chromosomes per subgenomes A, B and D, all of which are present as duplicate sets (AABBDD). As a result, the wheat genome is very large with about 15 Gb containing more than 85 % of repetitive DNA and it has only been fully sequenced in recent years (International Wheat Genome Sequencing Consortium, 2018). One consequence is that the wheat proteome is also complex. First, gluten proteins have an imbalanced amino acid composition with 11–29 % of proline and 32–53 % of glutamine, which are arranged in repetitive sequences with high sequence homology and only certain amino acid substitutions, deletions or insertions (Wieser et al., 2022; Shewry and Belton, 2024). On the opposite, they contain few amino acids with charged side chains (2–7 %) (Wieser et al., 2022). Furthermore, the special amino acid composition and repetitive character of gluten proteins pose a challenge for shotgun (bottom-up) proteomics since it is difficult to identify unique peptides allowing a distinction of individual gluten proteins. In bottom-up proteomics, proteins are first hydrolyzed into peptides by peptidases instead of being measured as intact proteins (top-down proteomics). The peptides are then separated using high-performance liquid chromatography (HPLC) and detected using mass spectrometry (MS) after ionization. This can be done, for example, by electrospray ionization (ESI). Subsequently, precursor ions are fragmented into product ions and analyzed by MS/MS. The resulting mass-to-charge ratios are matched to amino acid sequences and subjected to a database search (e.g. UniprotKB), allowing for an assignment of peptides to proteins (Tyanova et al., 2016; Alves et al., 2019). As already mentioned, this is challenging with gluten peptides because they are so similar. One solution is to generate as many peptides as possible with properties that are suitable for proteomics measurements. This is the only way to achieve an accurate determination of the proteins with a high sequence coverage.

The enzyme trypsin is used as standard for the digestion of proteins in proteomics workflows. It has also recently been used to study the proteome of wheat species (Afzal et al., 2023). Trypsin is a very efficient and specific serine endopeptidase, which cleaves after the basic amino acids lysine and arginine. Tryptic peptides therefore contain at least one basic amino acid and are well suited for ESI in the positive mode as they can be easily protonated (Jaskolla et al., 2009). However, protein digestion with only one peptidase will lead to an incomplete and biased proteome (Tsiatsiani and Heck, 2015). Sufficient protein sequence coverage is also not always achieved. There are many other proteolytic enzymes as possible alternatives to trypsin including chymotrypsin, Lys-C, Lys-N, Asp-N, Glu-C and Arg-C. The use of multiple peptidases led to 18 % more protein identifications and better protein sequence coverage compared to trypsin alone (Swaney et al., 2010). Using more than one peptidase can also be an advantage when searching for specific proteins or sequence domains. For example, Giansanti et al. (2015) have shown that phosphorylation sites can be linked to specific peptidases increasing their detectability by a factor of 1000. Regarding label-free absolute quantification, there is also a bias depending on the peptidase used (Giansanti et al., 2015; Peng et al., 2012).

Furthermore, it has already been established that some gluten proteins lack possible cleavage sites for trypsin (Ferranti et al., 2007; Dupont et al., 2011). ω-Gliadins in particular contain 20–30 % of proline (Scherf, 2023), which interferes with tryptic digestion, because it usually does not cleave when lysine or arginine is followed by proline. This raises the question whether the resulting peptides are suitable for analysis by liquid chromatography tandem mass spectrometry (LC-MS/MS) and how extensive the protein coverage is. It is therefore important to test other peptidases or peptidase combinations besides trypsin.

Chymotrypsin has already been used more frequently in context of gluten proteins (Colgrave et al., 2017; Manfredi et al., 2015; Fiedler et al., 2014; Norwig et al., 2024; Vensel et al., 2011). It cleaves after phenylalanine, tryptophan and tyrosine, and less specific after leucine and methionine. However, like trypsin, proline at the P1’ position prevents cleavage by chymotrypsin. Due to its specificity, chymotrypsin forms complementary peptides to trypsin, making it interesting for both single and combined digestion with trypsin. Pepsin cleaves at different sites depending on the pH value. At pH 1.3, it cleaves before and after phenylalanine and leucine. At pH values >2, it additionally cleaves before and after tryptophan and tyrosine. Since protein extraction is typically carried out at pH 6–8, a subsequent acidification represents an additional processing step. Pepsin has been used to investigate barley (Colgrave et al., 2017) and durum wheat proteins (Prandi et al., 2012) and it is employed together with trypsin and chymotrypsin in all studies that aim to mimic human gastrointestinal digestion (Ogilvie et al., 2021; Lexhaller et al., 2020). Thermolysin has also already been used to investigate the wheat proteome (Dupont et al., 2011; Vensel et al., 2014) and to elucidate the intermolecular disulfide bonds between gluten proteins (Lutz et al., 2012). Thermolysin cleaves before alanine, phenylalanine, isoleucine, lysine, methionine and valine but not after aspartic and glutamic acid. This means that it has the highest number of potential cleavage sites considering all four enzymes mentioned here, which could be an advantage for gluten proteins.

For this study, flour extracts of nine common wheat genotypes were digested with four different peptidases and one combination: trypsin, chymotrypsin, pepsin, thermolysin and the combination of trypsin and chymotrypsin (TC). All these enzymes or combinations were used in the past but in various studies and with different sample sets or grain types. The aim of this study was therefore to provide a better overview and comprehensive comparison of the digestibility of wheat proteins – in particular gluten proteins – with different peptidases. The higher number of peptides generated by various enzymes compared to using only one enzyme may improve the determination and sequence coverage of gluten proteins. This is a prerequisite for fully mapping the wheat proteome in the future and linking genetic information with protein data.

## 2 Materials and methods

### 2.1 Chemicals

2-Chloroacetamide (98 %) was obtained from Acros Organics (Geel, Belgium). Tris(2-carboxyethyl)phosphine hydrochloride (98 %) was obtained from Alfa Aesar (Ward Hill, MA, USA). 1,4-Dithiothreitol (min. 99.5 %) was obtained from Applichem (Darmstadt, Germany). Ammonium bicarbonate (NH_4_HCO_3_, 100 %) and tris(hydroxymethyl)aminomethane hydrochloride (TRIS-HCl, ≥99 %) were obtained from Carl Roth (Karlsruhe, Germany). Acetonitrile (MS-grade, 100 %), formic acid (MS-grade, ≥99.9 %) and water (MS-grade, 100 %) were obtained from Thermo Fisher Scientific (Waltham, MA, USA). 1-Propanol (100 %), disodium hydrogen phosphate dihydrate (Na_2_HPO_4_ × 2 H_2_O, 99.9 %), ethanol abs. (100 %), potassium hydrogen phosphate (KH_2_PO_4_, 99.9 %), sodium chloride (NaCl, 99.9 %) and urea (>99.5 %) were obtained from VWR Chemicals (Radnor, PA, USA).

Trypsin (from bovine pancreas, TPCK-treated, enzyme activity according to the manufacturer: ≥10,000 U/mg protein), α-chymotrypsin (from bovine pancreas, TPCK-treated, enzyme activity according to the manufacturer: ≥40 U/mg protein), thermolysin (from *Geobacillus stearothermophilus*, enzyme activity according to the manufacturer: 30–350 U/mg protein) and pepsin (from porcine gastric mucosa, enzyme activity according to the manufacturer: ≥250 U/mg protein) were from Sigma-Aldrich (St. Louis, MO, USA).

### 2.2 Wheat flours

Wheat flours of nine wheat cultivars and breeding lines were used: Ambition, FIRL3565, Bussard, Event, Format, Julius, BAYP4535, Potenzial and RGT Reform. Except for RGT Reform, these genotypes represent the parental lines of the Bavarian Multiparent Advanced Generation Intercross (MAGIC) Wheat population (BMWpop), which was developed at LfL (Stadlmeier et al., 2018). RGT Reform is currently one of the most commonly grown cultivars in Germany and serves as a comparative standard. All nine genotypes were grown in the same field trial, which was carried out by Strube D&S GmbH in Söllingen, Germany, in 2018. Field management followed the recommended agricultural practices. Grain samples were mixed in equal proportions from four to 18 randomized 6 m^2^ plots for each genotype. Grains were cleaned using a 2.2 mm sieve and milled using a Bühler MLU-202 laboratory mill (Bühler AG, Uzwil, Switzerland), resulting in flours of type 550 according to the German flour classification system (ash content of 0.51–0.63 % based on dry matter).

### 2.3 RP-HPLC-UV measurement

Flour (100 mg) was weighed in triplicate. The following stepwise Osborne fractionation and RP-HPLC-UV measurement were done exactly as reported in Jahn et al. (2024).

### 2.4 LC-MS/MS measurement

#### 2.4.1 Protein extraction

The extraction was carried out based on the stepwise Osborne fractionation using three different extraction solutions: extraction solution 1 (ES1 for salt-soluble proteins) consisting of 0.07 mol/L Na_2_HPO_4_ × H_2_O and 0.4 mol/L NaCl which was adjusted to a pH of 7.6 with a solution containing 0.07 mol/L KH_2_PO_4_ and 0.4 mol/L NaCl, extraction solution 2 (ES2 for alcohol-soluble proteins) consisting of 60 % (v/v) ethanol in water and extraction solution 3 (ES3 for alcohol-insoluble proteins) consisting of 50 % (v/v) 1-propanol in 2 mol/L urea and 0.1 mol/L TRIS-HCl (pH 7.5) plus 1 % (w/v) dithiothreitol.

Flour (50 mg) was weighed in triplicate into a 2 mL microtube. ES1 (1 mL) was added followed by vortex mixing for 2 min. The tubes were sonicated for 5 min at 22 °C, incubated in a thermomixer (1500 rpm, 10 min, 22 °C) and centrifuged for 15 min at 21,380 rcf (relative centrifugal field, corresponds to standard acceleration of gravity *g*) and 22 °C. The supernatant was collected in a 2 mL microtube and evaporated to dryness (8 mbar, 4–6 h, 40 °C). To the residual pellet, 1 mL of ES2 was added and vortexed for 2 min. The tubes were sonicated for 5 min at 22 °C and incubated in a thermomixer (1500 rpm, 10 min, 22 °C). The tubes were centrifuged for 15 min at 21380 rcf and 22 °C. The supernatant was collected in a 2 mL microtube and evaporated to dryness (8 mbar, 4–6 h, 40 °C). To the residual pellet, 1 mL of ES3 was added and vortexed for 2 min. The tubes were sonicated for 5 min at 22 °C and incubated in a thermomixer (1500 rpm, 30 min, 60 °C). The tubes were centrifuged for 15 min at 21380 rcf and 22 °C. The supernatant was collected in a 2 mL microtube and evaporated to dryness (8 mbar, 4–6 h, 40 °C).

#### 2.4.2 Protein reduction and alkylation

The extracted proteins (ES1–3; n = 3 each) were dissolved in 300 μL of 0.5 mol/L TRIS-HCl (pH 8.5) and 300 μL of 1-propanol. For reduction, 100 μL of 0.05 mol/L tris(2-carboxyethyl)phosphine in 0.5 mol/L TRIS-HCl (pH 8.5) were added. The tubes were incubated in a thermomixer (1000 rpm, 30 min, 60 °C). For alkylation, 100 μL of 0.05 mol/L 2-chloroacetamide in 0.5 mol/L TRIS-HCl (pH 8.5) were added. The tubes were incubated in a thermomixer in the dark (1000 rpm, 45 min, 37 °C) and the solvent was evaporated to dryness (8 mbar, 4–6 h, 40 °C).

#### 2.4.3 Protein digestion

The reduced and alkylated protein extracts were dissolved in 800 μL of 0.1 mol/L TRIS-HCl (pH 7.8) and 0.04 mol/L urea. Depending on the experiment, trypsin, chymotrypsin, pepsin, thermolysin or the combination TC was used. The corresponding peptidase was dissolved at a concentration of 1 mg/mL and added to the extracts with an enzyme:substrate ratio of 1:50 (to calculate this, the protein content in E1-E3 was previously determined with RP-HPLC-UV). For thermolysin digestion, 0.5 mmol/L CaCl_2_ were added for enzyme activation. The tubes were incubated in a thermomixer in the dark (200 rpm, 37 °C, 18 h) for trypsin, chymotrypsin, thermolysin and TC.

For the digestion with pepsin, the samples were dissolved in 800 μL of 0.15 mol/L HCl to obtain a pH value < 2 and added at the same ratio as described above. The tubes were incubated in a thermomixer in the dark (200 rpm, 37 °C, 4 h).

The digestive action of all enzymes was stopped by heating the tubes at 95 °C for 5 min. The extracts were then purified directly with solid phase extraction (SPE).

#### 2.4.4 Solid phase extraction

SPE was done using Discovery DSC-18 SPE 96-well plates with a bed weight of 100 mg/well (Supelco, Sigma-Aldrich). The wells were activated with 2 mL of methanol and equilibrated with 2 mL of 80 % (v/v) acetonitrile and 0.1 % (v/v) formic acid in water. Afterwards, a conditioning step was performed with 3 mL of 2 % (v/v) acetonitrile and 0.1 % (v/v) formic acid in water. The digested samples were loaded onto the wells and allowed to drip through without vacuum. Then, they were washed with 5 mL of 2 % (v/v) acetonitrile and 0.1 % (v/v) formic acid in water. The peptides were eluted without vacuum with 2 × 0.5 mL of 40 % (v/v) acetonitrile and 0.1 % (v/v) formic acid in water. The eluates were collected in 2 mL microtubes and the solvent was evaporated to dryness (8 mbar, 4–6 h, 40 °C).

#### 2.4.5 LC-MS/MS measurement

The purified samples were reconstituted in 1 mL of 2 % acetonitrile and 0.1 % formic acid (ES1–2) or 0.5 mL of 2 % acetonitrile and 0.1 % formic acid (ES3). The LC-MS/MS measurements were performed on a Vanquish U-HPLC (Thermo Fisher Scientific, Waltham, MA, USA) coupled to an Orbitrap Q Exactive plus MS/MS system (Thermo Fisher Scientific).

The peptides (injection volume of 20 μL) were separated on an Aeris PEPTIDE XB-C18 (1.7 μm, 10 nm, 150 mm × 2.1 mm) LC column (Phenomenex, Torrance, CA, USA) at a flow rate of 0.2 mL/min. The solvents used were (A) 0.1 % formic acid in water and (B) 0.1 % formic acid in acetonitrile. The linear gradient was set as follows: 2 % B at 0 min, 10 % B at 1 min, 30 % B at 18 min, 40 % B at 21 min, 60 % B at 23 min, 80 % B for 25–27 min and 2 % B for 28–35 min. The eluent from U-HPLC was directly coupled to the ESI source of the Q Exactive plus MS/MS systems. The ion spray voltage was set to 3.0 kV, the sheath gas flow rate to 35 and the auxiliary gas flow rate to 10. No sweep gas was used. The capillary temperature was set to 350 °C and the radio frequency level of the S-lens was 60.

Data were acquired in data-dependent acquisition (DDA) and in positive ESI mode. Full MS parameters were set as follows: resolution: 70,000, automated gain control (AGC): 3e6, maximum injection time (IT): 50 ms, scan range: *m/z* 360–1300. The parameters for dd-MS^2^-scans were set as follows: resolution: 17,500, AGC target: 1e5, maximum IT: 100 ms, TopN: 10, isolation window: *m/z* 2.0, fixed first mass: *m/z* 120.0, normalized collision energy: 28. Dynamic ion exclusion was set to 20 s. The method runtime was 35 min.

#### 2.4.6 Protein identification

The MaxQuant software (version 2.4.9.0) with the integrated Andromeda search engine was used for protein identification (Tyanova et al., 2016). The MS/MS raw data were searched against a wheat protein database from UniProtKB (fasta file for organism_id [4565], downloaded on Sept. 28, 2023 containing 151,978 protein entries). The default settings of MaxQuant were retained except for the following changes. Oxidation of methionine and N-terminal protein acetylation were set as variable modifications and carbamidomethylation on cysteines as fixed modifications Label-free quantification (LFQ) was selected and the default settings kept. Trypsin, chymotrypsin, thermolysin, pepsin (pH < 2) and the combination TC (both peptidases were selected in the MaxQuant run) were selected as used in the experiment. Match between runs was enabled. The MaxQuant evaluations were done separately for each enzyme. For a more detailed examination, the results of all enzymes were evaluated in one MaxQuant run for the cultivar RGT Reform.

### 2.5 Statistical analysis

All experiments were carried out as three technical replicates. Data analysis and statistics were performed with Excel, version 2016 (Microsoft, Redmond, WA, USA) and OriginPro, version 2023 (OriginLab Corporation, Northampton, MA, USA). Protein groups that were a potential contaminant were excluded. Protein groups were filtered according to their fasta header. For the visualization of the relative abundance of the gluten proteins, protein groups had to be identified in at least two of the three technical replicates. If a protein group was only found in one of the three replicates, this protein group was systematically filtered out and not taken into account. The means of the LFQ intensities were calculated for the replicates. The visualization of protein groups shared between the enzymes was performed with the Venn Diagram App (version 1.10) implemented in OriginPro. The heatmap was created with the Heat Map with Dendrogram App (version 2.00) implemented in OriginPro.

## 3 Results & discussion

### 3.1 Absolute number of identified peptides

**Fig. 1:**
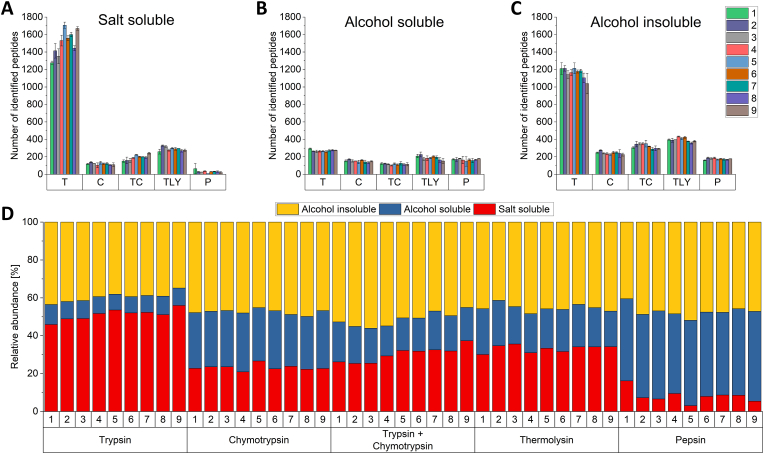
Total number of peptides identified per wheat genotype 1 to 9 and peptidase used in the salt-soluble fraction (A), in the alcohol-soluble fraction (B) and in the alcohol-insoluble fraction (C). The relative abundance of identified peptides between the three fractions is shown in panel D. 1: Ambition; 2: FIRL3565; 3: Bussard; 4: Event; 5: Format; 6: Julius; 7: BAYP4535; 8: Potenzial; 9: RGT Reform; T: trypsin; C: chymotrypsin; TC: trypsin + chymotrypsin; TLY: thermolysin; P: pepsin.

Looking first at the number of peptide identifications, most peptides were identified after trypsin digestion in all three extracts ES1–ES3 (Fig. 1 A–C). Particularly in the salt-soluble and alcohol-insoluble fractions, there was a major difference to the other digestive enzymes. All enzymes delivered similar results only in the alcohol-soluble fraction.

On closer examination of the salt-soluble fraction (Fig. 1 A), 1250 to 1700 peptides were identified in the wheat genotypes after trypsin digestion. This fraction also showed the greatest variation between the individual nine wheat genotypes. A total of 250–350 peptides were identified after thermolysin digestion, which is far less than after trypsin digestion. CT digestion resulted in 150–250 peptides, followed by chymotrypsin digestion with 100–130 peptides. Pepsin digestion only led to 30 to 60 peptides. The results clearly showed that trypsin was best at cleaving the proteins into identifiable peptides, resulting in 1000 more identifications compared to any other enzyme tested. Pepsin did not seem to be suited for analyzing the salt-soluble fraction at all as only few peptides could be identified (60).

In case of the alcohol-soluble fraction (Fig. 1 B), similar peptide numbers were obtained with all enzymes. In total, however, only 100 to 300 peptides were identified. Most peptides were detected with trypsin (250 to almost 300). Again, thermolysin followed with 150–200 peptides. Furthermore, 150 to 180 peptides were detected with pepsin. About 130 to 160 peptides were identified with chymotrypsin, while CT digestion resulted in 100–130 peptides. Since a rather small number of peptides was identified in the alcohol-soluble fraction with each enzyme, this fraction appears to be “difficult to digest”. None of the enzymes seems to be well suited to analyze the alcohol-soluble fraction. Alternatively, this could indicate that not many different proteins are present in this fraction.

The alcohol-insoluble fraction (Fig. 1 C) shows a similar pattern to the salt-soluble fraction. Between 1100 and 1250 peptides were identified after trypsin digestion. Thermolysin digestion led to the detection of 350–400 peptides. After chymotrypsin digestion, there were about 190–280 peptides, and after combined CT digestion, 290 to 370 peptides were identified. Pepsin had the lowest number of identifications with 150–200 peptides. As with the salt-soluble fraction, trypsin appears to be most suitable for analyzing the alcohol-insoluble fraction, because of the much higher number of peptides identified.

Across all nine wheat genotypes and all three fractions, a total of 4115 different peptides were identified with trypsin. With thermolysin, we identified 1421 peptides in total, followed by TC with 1141, chymotrypsin with 910 and pepsin with 606 peptides. In summary, trypsin was most suitable to obtain wheat peptides that can be identified by LC-MS/MS. All other peptidases used have a higher number of possible cleavage sites. This could lead to shorter peptides and if peptides are too short, they become unsuitable for identification by LC-MS/MS, because they are too unspecific to be assigned to the corresponding protein of origin. Therefore, a peptide length of 7–25 amino acids is considered to be best. Peptides that are too short have already been observed in chymotryptic digests of gluten proteins (Colgrave et al., 2017). Even after trypsin digestion, it has been reported that more than half of the peptides were less than six amino acids long and therefore difficult to identify (Tsiatsiani and Heck, 2015; Swaney et al., 2010). However, these findings were based on yeast peptides and do not necessarily apply to wheat peptides. Especially the peptides belonging to gluten proteins often contain repetitive sequences consisting mainly of proline and glutamine with very few lysine and arginine residues. Consequently, there are not as many cleavage options for trypsin. The results obtained here confirm this: after trypsin digestion, the average gluten peptide length was 21 amino acids. After pepsin and thermolysin digestion, the peptides averaged 14 and 15 amino acids in length. For chymotrypsin and CT, the average peptide length was 16 amino acids each. In addition, most peptides with a length of over 30 amino acids were measured with trypsin: 59 in contrast to a maximum of 21 for the other peptidases (Fig. S1). Most gluten peptides were identified with thermolysin (814), followed by chymotrypsin (447), TC (444), trypsin (365) and then pepsin (368). This is in line with literature, where most gluten peptides were also detected with thermolysin, followed by chymotrypsin and then trypsin (Vensel et al., 2011). A count of 434 wheat gluten peptides was identified after sequential digestion with chymotrypsin and trypsin and the combination of these results with the results of sequential digestion with Lys-C and trypsin (Martinez-Esteso et al., 2016). This result of a sequential digestion is also very close to our result of combined digestion even if no Lys-C was used in our study. It is noticeable that the order of the peptidases changes if only the amount of gluten peptides and not all peptides present in the extracts are considered.

Another important peptidase characteristic, also for targeted proteomics, is the complete and reproducible cleavage of the proteins into peptides, i.e., that there are no missed cleavages. Among the tryptic peptides, 1210 had missed cleavages, which corresponds to 29 %. This shows that trypsin not only led to by far the most peptide identifications, but also appears to cleave most completely in comparison to the other peptidases. This confirms that trypsin is a very efficient and specific peptidase (Tsiatsiani and Heck, 2015). After thermolysin digestion, 83 % of the peptides showed missed cleavages possibly due to its many cleavage sites. With 47 %, almost half of all peptides had missed cleavages after the combined TC digestion and 39 % of the peptides were not completely cleaved during digestion with chymotrypsin. That chymotrypsin does not cleave as specific as trypsin was also noted before (Colgrave et al., 2017). After pepsin digestion, 86 % of the peptides contained missed cleavages, which may also be due to the many cleavage sites of pepsin and the fact that it cleaves in a strongly pH-dependent manner. The less specific the enzyme is, the more cleavage sites there are, but the greater the probability of missed cleavages (Jaskolla et al., 2009). The results obtained in the present study confirm this.

### 3.2 Relative abundance of identified peptides

As shown in Fig. 1 D, the relative abundance of peptide identifications between the fractions varies depending on the peptidase. After trypsin digestion, 46–56 % of the peptides were detected in the salt-soluble fraction, while 35–44 % were identified in the alcohol-insoluble fraction. It is striking that only 9–11 % of the peptides belonged to the alcohol-soluble fraction. As already discussed, this is likely due to the presence of similar proteins in this fraction and the lack of suitable cleavage sites for trypsin. The digestion with chymotrypsin, TC and thermolysin showed similar distributions: most of the peptides (41–46 %) were identified in the alcohol-insoluble fraction. A similar proportion of peptides was detected in both the salt-soluble (21–37 %) and alcohol-soluble fractions (16–31 %), with slight differences between the peptidases. In contrast, a similar number of peptides was identified in the alcohol-soluble (42–47 %) and insoluble fractions (40–48 %) after pepsin digestion, but far less in the salt-soluble fraction (3–16 %). Therefore, pepsin is not well suited to digest the proteins in the salt-soluble fraction under the selected conditions. The reasons for this different distribution have already been discussed in the previous chapter. Overall, the peptidases generate a highly variable number of peptides per fraction due to their different cleavage sites. This already indicates at the peptide level that the wheat proteome is represented differently by each peptidase.

### 3.3 Number of protein groups

The extraction was carried out in three consecutive steps with different extraction solutions in order to achieve comprehensive coverage of the proteome. Furthermore, this type of extraction is widely used for wheat flour, especially to quantify the different gluten protein types using RP-HPLC-UV (Xhaferaj and Scherf, 2024; Wieser et al., 1998; Schalk et al., 2017). Therefore, different proteins should be enriched in each fraction. The salt-soluble fraction should mainly contain albumins and globulins, which are metabolic proteins (Wieser et al., 2014). However, a closer look at the protein groups after trypsin digestion revealed that 31 of 701 identified protein groups belonged to gliadins and 18 to glutenins. This indicates that gliadins and glutenins are already extracted during the first extraction step. The alcohol-soluble fraction should mainly contain wheat storage proteins, namely the monomeric gliadins. In addition to gliadins, 30 glutenins were also detected in the alcohol-soluble fraction after trypsin digestion. Considering that a total of 60 gluten protein groups was identified, almost all of them are already found in this fraction. It has already been reported that monomeric glutenins can also be extracted from the flour with aqueous alcohol solutions (Rombouts et al., 2012). The alcohol-insoluble fraction is meant to contain the large and complex glutenins. Since a strong extracting agent is used and it is the third extraction step, this fraction also contains all kinds of other proteins that have not been extracted earlier. So all three fractions overlap in their protein groups which is in line with earlier reports (Fallahbaghery et al., 2017). In addition, further separation by preparative HPLC still did not lead to pure protein fractions containing only certain protein groups (Lexhaller et al., 2019).

**Fig. 2:**
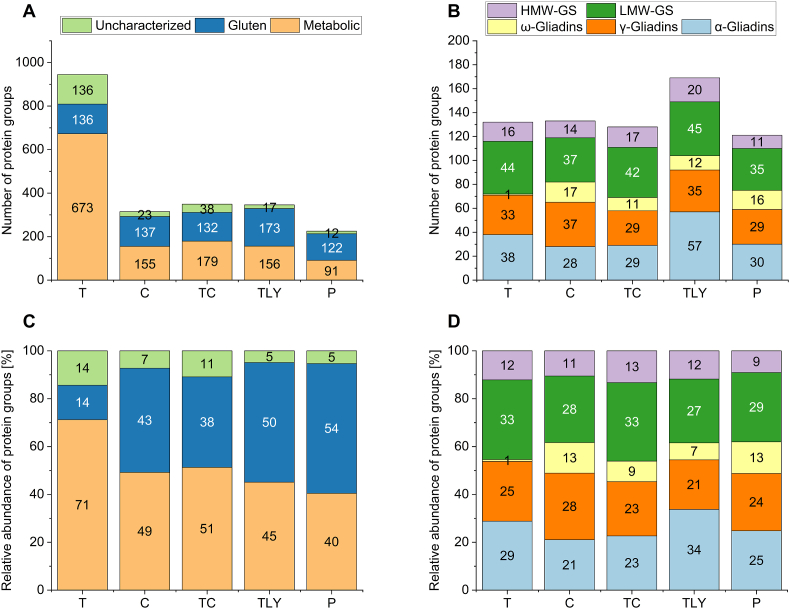
Absolute (A) and relative (C) abundance of metabolic, gluten and uncharacterized protein groups. Absolute (B) and relative (D) abundance of specific gluten protein groups. The results of all nine wheat genotypes were summarized for each peptidase used. Protein groups were identified at 1 % false discovery rate. T: trypsin; C: chymotrypsin; TC: trypsin + chymotrypsin; TLY: thermolysin; P: pepsin; HMW-GS: high-molecular-weight glutenin subunit; LMW-GS: low-molecular-weight glutenin subunit.

Consequently, all three fractions of the multi-stage extraction were evaluated together, especially with regard to quantitative evaluation. A total of 945 protein groups were detected in the nine wheat genotypes after trypsin digestion (Fig. 2 A). As expected, this was again by far the highest value compared to the other enzymatic digests. 349 protein groups were identified after the combined TC digestion and 346 and 315 protein groups after thermolysin and chymotrypsin digestion, respectively. With pepsin, only 225 protein groups were assigned, which can be explained by the small number of identified peptides. As already seen with the peptide identifications, the great influence of the peptidase is also evident here.

Since the protein groups are no longer divided into the three fractions according to solubility, they were divided according to their functions into metabolic, gluten and uncharacterized protein groups (Fig. 2 A). The largest difference between the peptidases was in the metabolic protein groups, because more metabolic protein groups were identified after trypsin digestion: 673 as opposed to 91–179 protein groups with the other enzymes/combination. In case of the most abundant proteins in wheat, the gluten proteins, around 130 gluten protein groups were identified with each enzyme. Only thermolysin stood out with 169 identified gluten protein groups. Between 50 and 100 gluten proteins are found in a single wheat genotype (Shewry and Belton, 2024). Vensel et al. (2011) identified 52 gluten proteins in one wheat cultivar (Butte 86) after combined evaluation of trypsin, chymotrypsin and thermolysin digestion. As this is a joint evaluation of nine wheat genotypes, these results are consistent with the literature. It should be noted that some protein groups remain uncharacterized due to missing information in the wheat protein database. In case of trypsin digestion, 136 protein groups were affected, which is 14 % of the total (Fig. 2 C). Another 14 % of all tryptic protein groups belong to gluten protein groups while the majority (71 %) are metabolic protein groups. For all other peptidases, the relative distribution was very similar: 40–51 % and 43–54 % are metabolic protein groups and gluten protein groups, respectively. The remaining protein groups (5–11 %) were uncharacterized.

As the gluten protein groups are among the most important proteins in wheat due to their role in bread making, the associated protein groups were broken down in more detail (Fig. 2 B and D). Every gluten protein type was identified with each enzyme. A total of 72 different gliadin protein groups were identified after trypsin digestion. After chymotrypsin digestion, there were 82 and after combined digestion, 69. In sum, 104 gliadin protein groups were found after thermolysin and 75 after pepsin digestion. Based on the total number of identified gliadin protein groups, it appears that more can be identified or differentiated (and thus categorized into more different groups) after chymotrypsin or thermolysin digestion. This may be an advantage if this fraction is to be examined in more detail.

Within the gliadin fraction, the number of protein groups assigned to α-gliadins varied from 28 to 57. Thermolysin in particular appears to be well suited to investigate α-gliadins, as most protein groups (57) were distinguished. The number of γ-gliadin protein groups varied from 29 to 37 and was therefore in a similar range for all peptidases. Most protein groups (37) were detected after chymotrypsin digestion. α- and γ-gliadins are present in roughly equal proportions in the total gluten content (Wieser et al., 2022). They also had a similar number of protein groups, as illustrated by the relative abundance of protein groups (Fig. 2 D). The identification of ω-gliadin protein groups constituted the biggest difference between the peptidases. Only one protein group could be assigned to the ω-gliadins using trypsin. ω-Gliadins consist of up to 80 % of the amino acids glutamine, proline and phenylalanine (Wieser, 2007). Since trypsin selectively cleaves after lysine and arginine, there may be simply not enough cleavage sites within the ω-gliadins. As a result, the peptides are too long to be identified with LC-MS/MS. All other peptidases used in this study also cleave before or after phenylalanine resulting in suitable ω-gliadin peptides that can be detected by LC-MS/MS. ω-Gliadins only make up around 10 % of gluten proteins (Geisslitz et al., 2018; Hajas et al., 2018), which may be why only few different ω-gliadins are found at all. ω-Gliadins only make up a small proportion of the gluten proteins and have not yet been linked to properties such as the baking quality of wheat. Nevertheless, if the ω-gliadin fraction is to be analyzed using bottom-up proteomics, it may be better to choose an enzyme other than trypsin for digestion, e. g., chymotrypsin.

Within the glutenin proteins, the LMW-GS were reported to have a greater variation than the HMW-GS (Shewry and Lafiandra, 2022). This is in line with our findings: 35–45 individual protein groups belonging to the LMW-GS were distinguished with every peptidase. Most protein groups (45) were detected after thermolysin digestion, but there were no large differences between the peptidases. In contrast to the LMW-GS (7–16), only 3–5 individual HMW-GS are known to be present per wheat genotype (Shewry and Lafiandra, 2022). Also here, only few different proteins were detected, but a total of 11–20 among the nine wheat genotypes. Again, most protein groups (20) were detected after thermolysin digestion. HMW-GS also contain a relatively high amount of glycine in the repetitive regions (Wieser, 2007). This basically simplifies the assignment of peptides to specific proteins. Furthermore, polar amino acids like lysine occur more frequently in the non-repetitive regions (Shewry and Belton, 2024). This is an advantage when using trypsin, as it selectively cleaves for lysine. However, this is not reflected in the number of protein groups, as similar results were obtained with all enzymes. In general, the results obtained here are reflected in the literature, which reports 20 different HMW-GS and over 40 different LMW-GS in wheat (Hu et al., 2023).

### 3.4 Relative abundance of gluten proteins

**Fig. 3:**
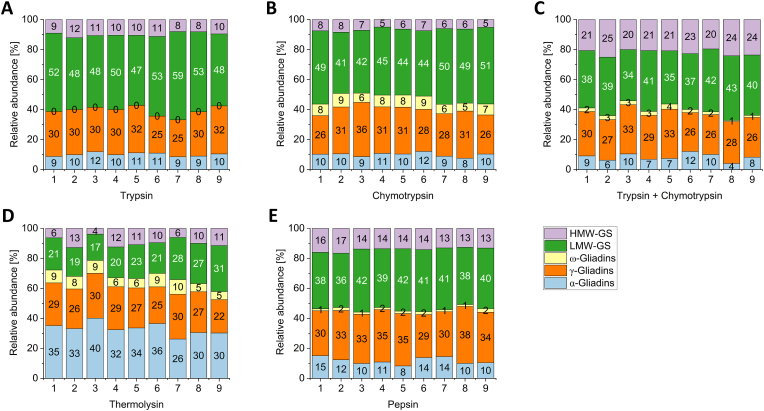
Relative abundance of gluten proteins in the investigated wheat genotypes based on the LFQ intensities of the associated protein groups. The abundance varies depending on the peptidase including trypsin (A), chymotrypsin (B), trypsin and chymotrypsin (C), thermolysin (D) and pepsin (E). LFQ: label-free quantification; 1: Ambition; 2: FIRL3565; 3: Bussard; 4: Event; 5: Format; 6: Julius; 7: BAYP4535; 8: Potenzial; 9: RGT Reform. HMW-GS: high-molecular-weight glutenin subunit; LMW-GS: low-molecular-weight glutenin subunit.

The total amount of gluten proteins in a flour can be determined using HPLC and an external standard (Wieser et al., 1998; Schalk et al., 2017). Consequently, we summed the LFQ intensities of all protein groups within the respective gluten protein type and determined the respective percentage share (Fig. 3). The pattern varied depending on the peptidase. The distribution pattern using trypsin, chymotrypsin, TC and also pepsin looked very similar: approx. 10 % of α-gliadins and 30–35 % of γ-gliadins were present followed by a smaller share (0–10 %) of ω-gliadins. Only one ω-gliadin was identified after trypsin digestion. However, it had an LFQ intensity of zero, so it was not taken into account. It has also been reported in the literature that no ω-gliadin peptides were detected after trypsin digestion (Vensel et al., 2011). The LMW-GS and HMW-GS were divided into 40–50 % and 10–20 %. Only the thermolysin digest showed a divergent pattern, which is mainly reflected in proportionally more α-gliadins (26–40 %) and less LMW-GS (17–31 %). Slightly fewer γ-gliadins were determined after thermolysin digestion (22–30 %). ω-Gliadins ranged from 5 % to 10 % and HMW-GS from 4 % to 11 %. This shows that the abundance highly depends on the peptidase. It also follows that the comparison of LFQ data of different peptidases is not possible. Instead, only data sets obtained with the same peptidase can be compared. This was also the conclusion reached by Giansanti et al. (2015) who investigated the human phosphoproteome with multiple proteases. Based on this, however, trypsin shows a distorted picture of the gluten proteins, because, e.g., the ω-gliadins were not found after trypsin digestion. This proteome bias has already been pointed out in other studies (Tsiatsiani and Heck, 2015; Peng et al., 2012; Giansanti et al., 2016). Even with absolute quantification approaches, the same peptides produced by different peptidases exhibit a bias. This was attributed to differences in digestion conditions, protein structure and the peptidase itself (Woessmann et al., 2022).

After RP-HPLC-UV analysis, the α-, γ- and ω-gliadins ranged from 25–32 %, 23–29 % and 7–11 % of total gluten, respectively. LMW-GS and HMW-GS ranged between 22–30 % and 8–12 % (Fig. S2). This is consistent with the literature, where α-, γ-, and ω-gliadins account for 35 %, 21 %, and 11 % of total gluten, respectively (Scherf, 2023). Only the LC-MS/MS results using thermolysin roughly corresponded to this distribution (Fig. 3). Particularly in the case of α-gliadins, there was a major difference between thermolysin and the other peptidases. More α-gliadin protein groups could be distinguished after thermolysin digestion and this is reflected here in a higher proportion of α-gliadins.

According to Scherf (2023), the HMW-GS have a share of 9 % and the LMW-GS a share of 24 %, which is also consistent with the RP-HPLC-UV results obtained here. Again, the results from LC-MS/MS using thermolysin corresponded to this. For the HMW-GS, the results of most peptidases were in a similar or slightly higher range. The LMW-GS showed the largest differences, because the share of 40–50 % was more than twice as high (the only exception is thermoylsin with LMW-GS proportions of 20–30 %). Since some LMW-GS are similar to the γ- and ω-gliadins (Shewry and Belton, 2024) it is possible that peptides that originally belonged to the γ- and ω-gliadins were assigned to the LMW-GS. The reason for this could lie in the short N- and C-terminal residues of the ω-gliadins, which only contain 10 to 20 amino acids (Shewry and Belton, 2024). If an enzyme does not cleave a characteristic terminal peptide, the corresponding protein is not found. This applies in particular to tryptic peptides. As a result, they may be assigned to the LMW-GS. This is also reflected in the relative amount of LMW-GS, which was highest for trypsin (Fig. 3). However, different gluten protein types are only assigned via their retention time in HPLC analyses and not with an exact identification as with LC-MS/MS. Consequently, HPLC analysis cannot differentiate between α-/γ-gliadins and LMW-GS. It has already been pointed out here that glutenins are already present in the alcohol-soluble fraction. Hence, they are also extracted there to a certain extent. This cannot be distinguished in the HPLC analyses and they are subsequently assigned to the gliadins. This also applies to the other two fractions, as various gluten proteins were also detected there with LC-MS/MS.

Overall, the glutenin protein fraction made up the largest proportion with 50–60 % for every peptidase but thermolysin, which is contrary to the results of Wieser (2000), where these have a proportion of 35 % in winter wheat. In another study in which the proteins were extracted with Osborne fractionation and determined by HPLC, the glutenins make up almost 30 % of the gluten proteins in common wheat (Geisslitz et al., 2018). This is half of the glutenin share calculated here. The gliadin-to-glutenin ratio was between 0.5 and 1.0 for all peptidases, except thermolysin, where it was between 1.4 and 3.6. This is in the same range as for the HPLC results in the literature, which where for example 2.0–3.2 (Geisslitz et al., 2018) or 2.1–4.1 (Hajas et al., 2018).

However, it remains exciting to see which results correspond most closely to the actual protein distribution in wheat – both qualitatively and quantitatively. Overall, there were variations between the individual wheat genotypes, both in their protein group composition and with regard to the respective quantity. This variation could be utilized within breeding programs (Bose et al., 2019; Afzal et al., 2021).

Taken together, the results underline the challenges of bottom-up proteomics of wheat storage proteins. They contain highly repetitive sequences that either do not have sufficient cleavage sites and/or result in similar peptides which can be assigned to several proteins. This suggests that some of the peptides were not assigned to their original proteins, but to proteins of other gluten protein types. This would lead to false positive results. However, HPLC analyses also do not provide exact results, since gluten proteins were identified in all three fractions with LC-MS/MS. Further, LC-MS/MS analysis leads to very different results between the peptidases. In general, the values for ω-gliadins and HMW-GS remained the most consistent across all peptidases and evaluations. This is possibly due to their characteristic phenylalanine (ω-gliadins) or glycine (HMW-GS) content, which makes it easier to clearly assign them (Shewry and Belton, 2024). However, the advantage of proteomics which allows the exact identification of molecules, cannot be fully exploited for gluten proteins. Therefore, in the next section, only one wheat genotype will be analyzed in more detail to find out to what extent the different peptidases complement each other or yield different results.

### 3.5 Protein identifications and sequence coverage in wheat cultivar RGT reform

**Fig. 4:**
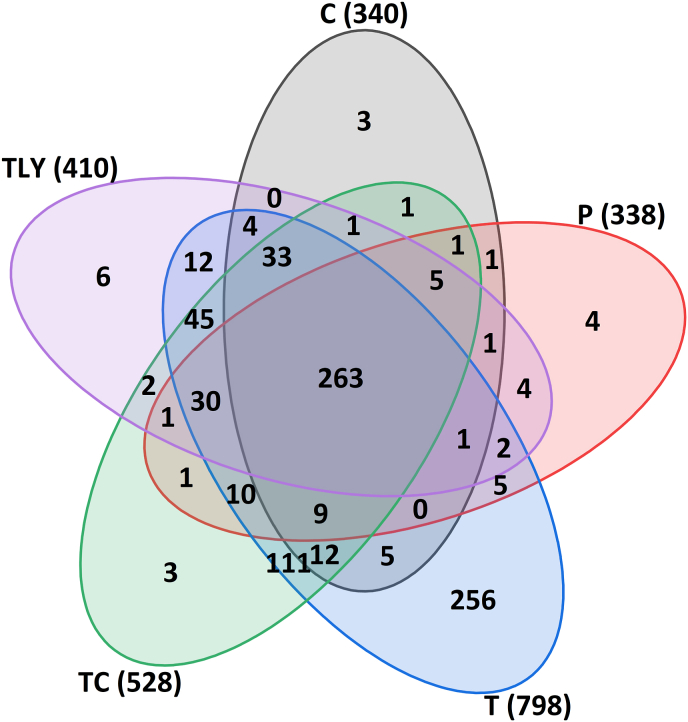
Number of protein groups in the wheat cultivar RGT Reform which are unique or common between the five different peptidases. The number in parentheses indicates the total number of protein groups found with the respective peptidase. T: trypsin; C: chymotrypsin; TC: trypsin + chymotrypsin; TLY: thermolysin; P: pepsin.

In this section, only the wheat cultivar RGT Reform is considered in more detail as a comparison standard for the parental wheat lines of the BMWpop (Stadlmeier et al., 2018). For this purpose, the measurements of all peptidases were evaluated in one MaxQuant run. We first compared which protein groups are assigned with each peptidase and the extent to which they overlap (Fig. 4). A total of 798 protein groups were identified with trypsin, whereas there were 528, 410, 340 and 338 protein groups with TC, thermolysin, chymotrypsin and pepsin, respectively. Of these protein groups, 263 were identified with all peptidases. Using trypsin, 256 protein groups were identified. For all others, only three to six protein groups were detected with only one peptidase.

Among the overlaps between the enzymes, trypsin and TC stood out in particular with 111 common protein groups. This is expected because trypsin is also used in the combination. The fact that chymotrypsin and TC only share one common protein group shows that trypsin is the more important actor in the combination. Trypsin, TC and thermolysin had 45 protein groups in common. If chymotrypsin is compared to trypsin, TC and thermolysin there were 33 common protein groups, while there were 30 with pepsin. All other overlaps were very low (0–12). The protein groups that were only found with trypsin are almost exclusively metabolic proteins, but also some uncharacterized proteins. The same applied to the 111 protein groups which were detected with trypsin and TC. These results illustrate once again that trypsin covers the proteome most comprehensively. The other peptidases provided almost no additional identifications of protein groups. The only exception was the ω-gliadins, which were detected with all peptidases, but not with trypsin. All other gluten protein types were detected with all peptidases. This is also reflected in the ten most abundant protein types obtained with the respective peptidase (Table S1). These were predominantly gluten proteins with proportions ranging from 2.3 to 24.1 %. In the case of trypsin, four of the ten most frequent proteins were metabolic proteins, mainly amylase/trypsin-inhibitors (ATIs) with proportions of 1.9–3.4 %. However, thermolysin and pepsin also have three metabolic proteins among the ten most abundant proteins with proportions of 2.0–7.3 %, while only one metabolic protein was identified more frequently with chymotrypsin and TC with a proportion of 13.8 % and 2.9 %, respectively. It is striking that ω-gliadins with proportions of 10.1–15.1 % are also among the ten most abundant proteins for all peptidases except trypsin. This once again underlines their better suitability for the identification of this specific gluten protein type. In contrast, trypsin appears to be particularly suitable for the identification of ATIs, where it has already been used successfully in targeted proteomics (Geisslitz et al., 2020; Jahn et al., 2025).

In general, a high specificity of the peptidase leads to more protein identifications. Peptidases that are not as specific, such as chymotrypsin, can benefit from pre-digestion with trypsin, leading to more protein identifications (Dau et al., 2020). This could not be confirmed here with TC digestion. One reason may be that the TC digestion did not take place one after the other, but simultaneously.

The average sequence coverage of gluten protein groups amounted to 61 %. In the experiments in which only one enzyme was evaluated, the average sequence coverage was 51 % for thermolysin. For TC, chymotrypsin and pepsin it was 37 %, 35 % and 30 %, respectively. It is interesting to note that the peptidase thermolysin and the combination TC, both of which had the most possible cleavage sites, gave the highest sequence coverage. Trypsin only resulted in a coverage of 27 %. In the literature, the highest sequence coverage was also found for thermolysin (between 31–49 %), followed by chymotrypsin (16–40 %) and trypsin (0–28 %). It was only for the γ-gliadins that chymotrypsin performed slightly better with 40 % than thermolysin with 35 % (Vensel et al., 2011). This confirms that, although trypsin cuts efficiently and specifically, it may not cover all regions of interest (Guo et al., 2014). Although the TC digestion did not result in more protein identifications, it led to a better gluten protein sequence coverage. Guo et al. (2014) also reported that the addition of double- and triple-enzyme digests did not lead to a major increase in protein identifications, but led to an improved mean sequence coverage from 33 % to 42 % (Guo et al., 2014). A multi-enzyme approach led to a modest increase in the protein groups, but a twofold improvement in proteome sequence coverage (Swaney et al., 2010). The higher protein coverage also indicates that the identified gluten proteins were assigned correctly with a higher probability.

**Fig. 5:**
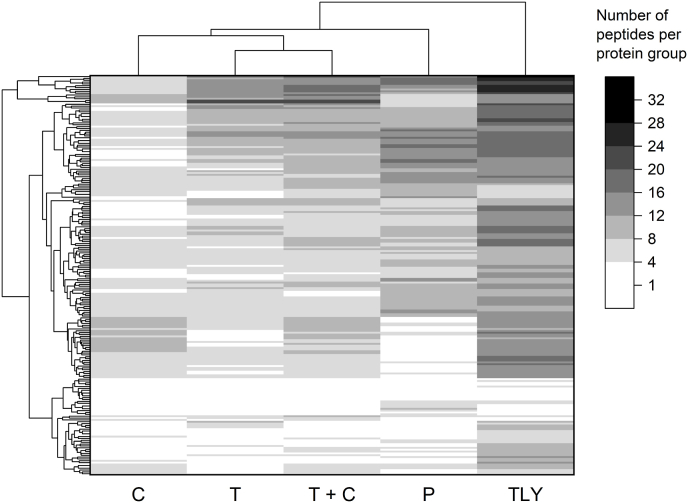
Heatmap based on the number of identified peptides per gluten protein group (215 in total). Shown are the bottom-up proteomics results for the wheat cultivar RGT Reform, which was digested with five different peptidases. White color means that no peptides were detected for this protein group. T: trypsin; C: chymotrypsin; TC: trypsin + chymotrypsin; TLY: thermolysin; P: pepsin.

Thermolysin achieved a comparatively high sequence coverage for the gluten proteins. To illustrate this, a heat map was generated which shows how many peptides were identified per gluten protein group (Fig. 5). Most peptides per protein group were identified with thermolysin. On average, there were twelve peptides per group which is also reflected very evenly in the heatmap. The groups with the highest number of peptide identifications were predominantly HMW-GS and LMW-GS. With TC and pepsin, there were seven peptides per protein group on average. Within the peptides detected with TC, there were particularly many HMW-GS in the protein groups containing the most peptides. With pepsin, there were predominantly LMW-GS and γ-gliadins. An average of six tryptic peptides were found per protein group, of which the groups with the most peptides were HMW-GS and LMW-GS. An average of four peptides per group were detected with chymotrypsin. Among the groups with the most peptide identifications, there were many α- and γ-gliadins. Even though trypsin was found to yield by far the most peptides (Fig. 1), this did not apply to gluten peptides.

## 4 Conclusions

By far the largest number of peptides was identified with trypsin. As a result, most protein groups could also be assigned. This was most noticeable in the metabolic proteins. In the gluten protein groups, however, a similar number of proteins was found with all peptidases. In addition, some proteins, such as ω-gliadins, were not detectable with trypsin under the used conditions. It is therefore necessary to use alternative peptidases or multiple peptidases to increase sequence coverage, as is the case with gluten proteins. These could be covered by up to 61 %, taking all peptidases into account. It should be emphasized that a coverage of 51 % was achieved with thermolysin, whereas only 27 % was achieved with trypsin. A higher sequence coverage can be advantageous if certain sequence regions are of interest or to identify suitable peptides for targeted proteomics. However, despite higher sequence coverage, it was not possible to compare the relatively quantified data of different proteins with each other. In comparison with HPLC results, thermolysin showed the best agreement. It will be interesting to see if further peptidases with different cleavage specificity or sequential digestion may provide a more comprehensive coverage of the wheat proteome in the future or whether top-down measurements will be an alternative. Overall, it remains challenging to distinguish individual gluten proteins via bottom-up proteomics.

## CRediT authorship contribution statement

**Christine Kaemper:** Conceptualization, Data curation, Formal analysis, Investigation, Methodology, Visualization, Writing – original draft. **Johanna Mossburger:** Formal analysis, Investigation, Writing – review & editing. **Manuel Geyer:** Project administration, Resources, Writing – review & editing. **Lorenz Hartl:** Funding acquisition, Project administration, Resources, Writing – review & editing. **Sabrina Geisslitz:** Conceptualization, Investigation, Methodology, Supervision, Writing – review & editing. **Katharina Anne Scherf:** Conceptualization, Funding acquisition, Project administration, Resources, Supervision, Writing – review & editing.

## Data availability

The processed data required to reproduce the above findings are available within the manuscript and its supplement. The mass spectrometry proteomics data have been deposited to the ProteomeXchange Consortium via the PRIDE (Deutsch et al., 2023; Perez-Riverol et al., 2025) partner repository with the dataset identifier PXD061314 (https://www.ebi.ac.uk/pride/archive/projects/PXD061314).

## Funding

The project was supported by funds of the 10.13039/501100005908Federal Ministry of Food and Agriculture (10.13039/501100005908BMEL) based on a decision of the Parliament of the Federal Republic of Germany via the 10.13039/501100010473Federal Office for Agriculture and Food (10.13039/501100010771BLE) under the innovation support programme. Project number 2818404G18 (BigBaking). Measurements at the Q Exactive Plus Orbitrap mass spectrometer were funded by the 10.13039/501100001659Deutsche Forschungsgemeinschaft (10.13039/501100001659DFG, 10.13039/501100001659German Research Foundation), project number 445432254. Co-funded by the European Union (ERC, GLUTENOMICS, 101040437). Views and opinions expressed are however those of the author(s) only and do not necessarily reflect those of the European Union or the European Research Council Executive Agency. Neither the European Union nor the granting authority can be held responsible for them. Open Access funding enabled and organized by Projekt DEAL.

## Declaration of competing interest

The authors declare that they have no known competing financial interests or personal relationships that could have appeared to influence the work reported in this paper.

## Abbreviations

ATI: amylase/trypsin-inhibitor
FDR: false discovery rate
HMW-GS: high-molecular-weight glutenin subunit
LFQ: label-free quantification
LMW-GS: low-molecular-weight glutenin subunit
C: chymotrypsin
P: pepsin
RP-HPLC-UV: reversed-phase high-performance liquid chromatography-ultraviolet
T: trypsin
TC: trypsin + chymotrypsin
TLY: thermolysin
