# Supplementary material for: Comparative shotgun proteomics analysis of wheat gluten proteins digested by various peptidases
Source: Curr Res Food Sci. 2025 May 26;11:101095. doi: 10.1016/j.crfs.2025.101095 (PMC12182313; doi:10.1016/j.crfs.2025.101095)
Supplement: Multimedia component 1 [file mmc1.pdf]

# Supplementary Material

# Comparative shotgun proteomics analysis of wheat gluten proteins digested by various peptidases

**Christine Kaemper[1], Johanna Mossburger[1,2,3], Manuel Geyer[4], Lorenz Hartl[4], Sabrina Geisslitz[3], Katharina Anne Scherf[3,5]***

[1] Department of Bioactive and Functional Food Chemistry, Institute of Applied Biosciences, Karlsruhe Institute of Technology (KIT), 76131 Karlsruhe, Germany

[2] Technical University of Munich, TUM School of Life Sciences, 85354 Freising, Germany

[3] Leibniz Institute for Food Systems Biology at the Technical University of Munich, 85354 Freising, Germany

[4] Bavarian State Research Center for Agriculture, Institute for Crop Science and Plant Breeding, 85354 Freising, Germany

[5] Technical University of Munich, TUM School of Life Sciences, Professorship of Food Biopolymer Systems, 85354 Freising, Germany

***Correspondence:*** Katharina Anne Scherf, Leibniz Institute for Food Systems Biology at the Technical University of Munich, Lise-Meitner-Str. 34, 85354 Freising, Germany, Email: k.scherf.leibniz-lsb@tum.de

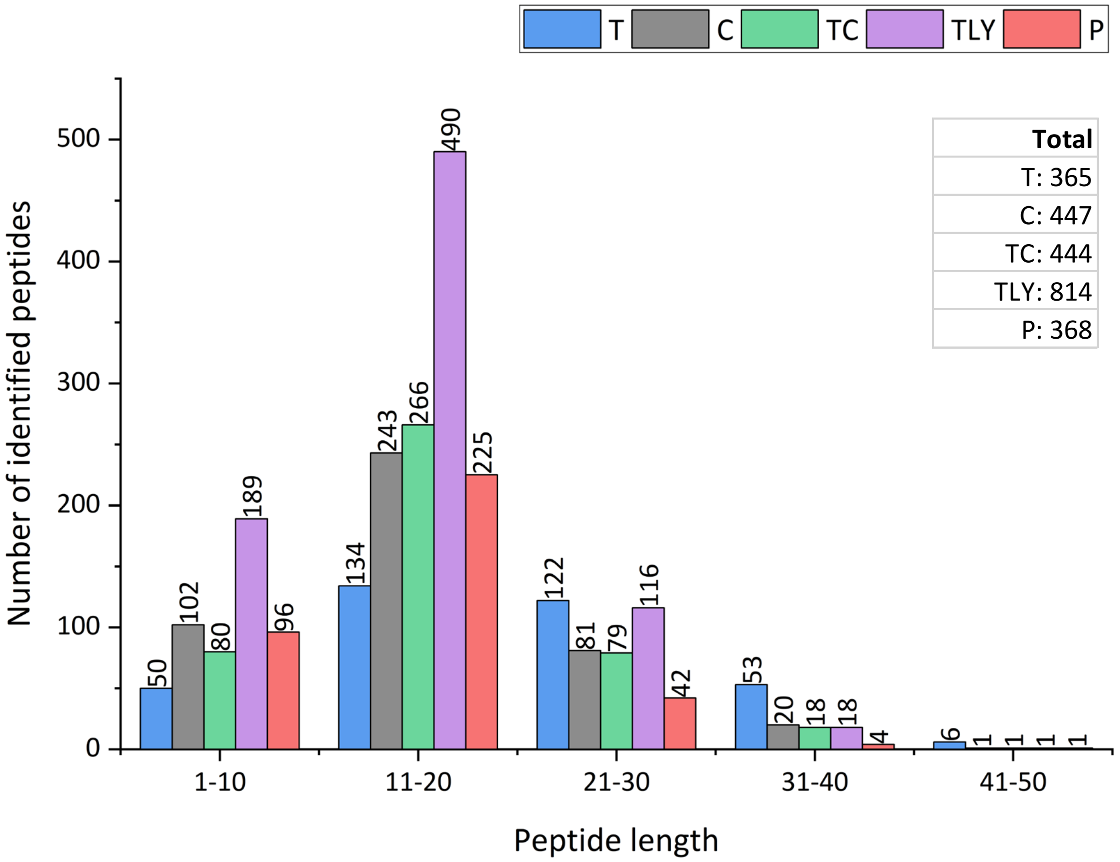


**Figure S1.** Distribution of gluten peptide lengths depending on each peptidase. The inset table on the right side indicates the total number of identified gluten peptides per peptidase. T: trypsin; C: chymotrypsin; TC: trypsin + chymotrypsin; TLY: thermolysin; P: pepsin.

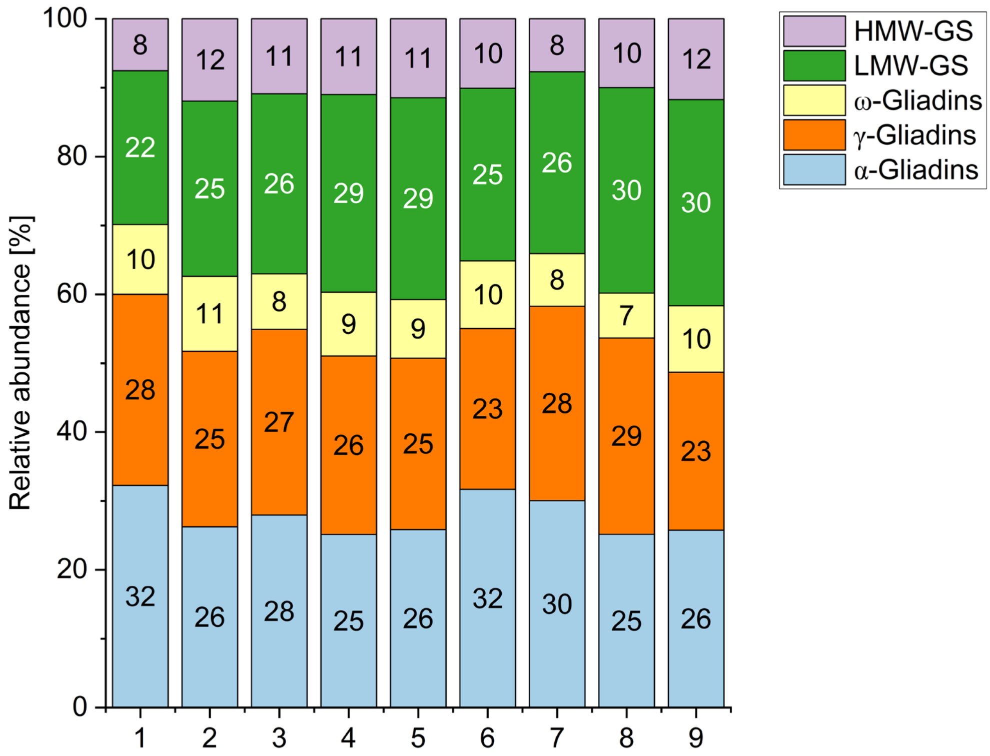


**Figure S2.** Relative abundance of gluten proteins in the investigated wheat cultivars based on RP-UHPLC-UV results of the intact proteins. 1: Ambition; 2: Firl3565; 3: Bussard; 4: Event; 5: Format; 6: Julius; 7: BAYP4535; 8: Potential; 9: RGT Reform. HMW-GS: high-molecular-weight glutenin subunit; LMW-GS: low-molecular-weight glutenin subunit.

**Table S1:** List of the ten most abundant protein types in wheat flour of cultivar RGT Reform obtained after digestion with different peptidases. A maximum of three protein IDs is listed under major protein ID and the relative proportion is indicated in parentheses following the respective protein type.

| Trypsin | | Chymotrypsin | | Trypsin + Chymotrypsin | | Thermolysin | | Pepsin | |
|---|---|---|---|---|---|---|---|---|---|
| Major protein ID | Protein type | Major protein ID | Protein type | Major protein ID | Protein type | Major protein ID | Protein type | Major protein ID | Protein type |
| B6UKN7 | γ-Gliadin (7.8%) | A9YSK5; Q42451; M4M8L5 | HMW-GS (14.4%) | A0A7D5DPJ7 | ω-Gliadin (10.1%) | A0A7D5DPJ7 | ω-Gliadin (11.1%) | B6UKN7 | LMW-GS (24.1%) |
| A0A2U8JCZ2; B2Y2Q1; R4JBL6 | LMW-GS (4.8%) | A0A3B6GZ31; A0A077RQJ1 | Calcyclin-binding protein (13.8%) | A0A2U8JCZ2; B2Y2Q1; R4JBL6 | LMW-GS (6.2%) | A9YSK5; Q42451; M4M8L5 | HMW-GS (8.0%) | A0A2U8JD23; M9TK56 | ω-Gliadin (15.1%) |
| Q5MD68; P01084Q4U1A4 | ATI (3.4%) | R4JFL5; R4JBH8; R4JB19 | LMW-GS (5.4%) | A9YSK5; Q42451; M4M8L5 | HMW-GS (6.0%) | R9XV26; A0A1K0J4W8 | α-Gliadin (7.6%) | Q5MD68; P01084; Q4U1A4 | HMW-GS (11.6%) |
| A0A3B6IJ76 | Cupin (3.4%) | A0A290XZ34 | γ-Gliadin (4.7%) | Q3S4V7 | α-Gliadin (5.7%) | X2KYP9; A0A7D5RE50; A0A7D5R7Y7 | ATI (7.3%) | A0A2U8JCZ2; B2Y2Q1; R4JBL6 | γ-Gliadin (9.4%) |
| A0A1S6KXP9 | ATI (2.7%) | P21292; R9XWA1; K7XEF1 | γ-Gliadin (4.4%) | R4JFL5; R4JBH8; R4JB19 | LMW-GS (5.4%) | A0A290XZ34 | γ-Gliadin (6.4%) | A0A3B6IJ76 | Pyruvat kinase (4.0%) |
| A0A060N0C7; V9P6D7; Q7X9J9 | LMW-GS (2.6%) | V9P6N2; Q5MFP8 | LMW-GS (4.2%) | A0A290XZ34 | γ-Gliadin (3.7%) | W5CQ97; A0A3B6GQ71; Q1XHC6 | Cystein proteinase (4.9%) | A0A1S6KXP9 | γ-Gliadin (3.6%) |
| A0A2U8JD23; M9TK56; Q30DX3 | γ-Gliadin (2.5%) | Q3S4V7 | α-Gliadin (4.0%) | R9XV26; A0A1K0J4W8 | α-Gliadin (2.9%) | A0A3B6I5M0 | Bi-functional inhibitor (4.2%) | P21292; R9XWA1K7XEF1 | α-Gliadin (2.8%) |

| | | | | | | | | | |
|---|---|---|---|---|---|---|---|---|---|
| R9XSX7 | α-Gliadin (2.0) | R9XV26; A0A1K0J4W8 | α-Gliadin (3.8%) | A0A3B6T7X8; Q7X9M4 | Bifunctional Inhibitor (2.9%) | Q5UHH8; Q5UHH6; Q4U1A1 | ATI (3.3%) | A0A060N0C7; V9P6D7; Q7X9J9 | α-Gliadin (2.7%) |
| P18573; J7HWD3; Q1WA39 | α-Gliadin (1.9) | A0A2U8JD23; M9TK56 | ω-Gliadin (3.7%) | Q41543; Q94G92; Q9M4L5 | γ-Gliadin (2.5%) | A0A0K2QJX0 | α-Gliadin (3.2%) | R9XSX7 | Grain softness protein (2.5%) |
| A4ZIZ6; A4ZIW9; A4ZIX1 | ATI (1.9%) | A0A7D5DPJ7 | ω-Gliadin (3.4%) | A0A060MZP1 | HMW-GS (2.3%) | A0A3B6T7X8; Q7X9M4 | Bifunctional inhibitor (2.9%) | P18573; J7HWD3; Q1WA39 | Serpin domain protein (2.0%) |

ATI, amylase/trypsin-inhibitor; HMW-GS, high-molecular-weight glutenin subunit; LMW-GS, low-molecular-weight glutenin subunit.
